# Stroke territory and atherosclerosis in ischemic stroke patients with a history of migraine with aura

**DOI:** 10.3389/fneur.2023.1142424

**Published:** 2023-02-27

**Authors:** Claudia Altamura, Giovanna Viticchi, Angelo Cascio Rizzo, Paola Maggio, Nicoletta Brunelli, Marilena Marcosano, Vincenzo Di Lazzaro, Fabrizio Fiacco, Elio Clemente Agostoni, Mauro Silvestrini, Fabrizio Vernieri

**Affiliations:** ^1^Department of Medicine and Surgery, Unit of Headache and Neurosonology, Università Campus Bio-Medico di Roma, Roma, Italy; ^2^Fondazione Policlinico Universitario Campus Bio-Medico, Roma, Italy; ^3^Neurological Clinic, Marche Polytechnic University, Ancona, Italy; ^4^Neurology and Stroke Unit, ASST Grande Ospedale Metropolitano Niguarda, Milan, Italy; ^5^Neurology Unit, ASST Bergamo Est, Seriate, Italy; ^6^Department of Medicine and Surgery, Unit of Neurology, Neurophysiology, Neurobiology, and Psychiatry, Università Campus Bio-Medico di Roma, Roma, Italy

**Keywords:** migraine aura, stroke, intima-media thickness, atherosclerosis, cerebral blood circulation

## Abstract

**Introduction:**

The mechanisms subtending the increased stroke risk in migraine with aura (MA) are not fully understood. Our study aims to evaluate if the clinical profile in stroke patients with MA differentiates from those without MA.

**Methods:**

We retrieved the prospective registered electronic clinical dossiers of adult patients younger than 60 years with acute ischemic stroke admitted in four hospitals between January 2016 and June 2022. Patients were classified by the history of MA (MA+ and MA–).

**Results:**

We identified 851 stroke patients (59 MA+, 6.9%). Compared to MA−, MA+ patients were characterized by younger age (44.0 ± 10.6 vs 50.1 ± 8.2 years), female sex (59.3% vs 29.0%), and affected by cryptogenic (OR 2.594 95% CI 1.483–4.537), and cerebellar stroke (OR 3.218 95% CI 1.657–6.250; *p* ≤ 0.001 for all comparisons). After adjusting for age and sex, MA+ patients presented less frequently hypertension (OR 0.349 95% CI 0.167–0.470; p=0.005) and dyslipidemia (OR 0.523 95% CI 0.280–0.974; *p* = 0.041). After adjusting also for risk factors, the MA+ group had less frequently symptomatic large vessel stenosis (OR 0.126 95% CI 0.017–0,924; *p* = 0.042) and clinical atherosclerosis (OR 0.103 95% CI 0.014–0.761; *p* = 0.026), while intima–media thickness did not differ (*p* = 0.395).

**Discussion:**

Cryptogenic and cerebellar stroke and fewer vascular risk factors and clinical atherosclerosis seem to characterize stroke patients with MA.

## Introduction

Migraine is one of the most prevalent and disabling neurological conditions worldwide (1). Recurrent headaches accompanied by typical symptoms such as nausea, noise, and light sensitivity allow migraine diagnosis to be made solely on clinical criteria (2). Approximately 30% of patients with migraine can experience transient neurological (i.e., visual, sensory, or aphasic) symptoms that characterize typical aura. While migraine can easily be diagnosed, its pathogenetic mechanisms are a complex entanglement that is not fully unraveled (3). These multifaceted mechanisms are also shared with a plethora of other clinical conditions which are comorbid with migraine (4). Among these disorders, acute vascular accidents are probably the most dreaded event in the lifetime of patients with migraine (5). Stroke risk is more consistent in patients with migraine with aura (MA), where, while the absolute risk of ischemic stroke is low, the relative risk is doubled when compared to the general population (6). Ischemic stroke in patients with MA can rarely occur in concomitance with an attack with a prolonged aura (i.e., migrainous infarction) (2), while most vascular events hit patients interictally.

Different pathogenetic mechanisms have been called into question to explain both conditions: a predisposing genetic background, thromboembolism, endothelial and hemodynamic dysfunction, and the inefficient use of energetic supplies (7, 8).

The increased prevalence and larger patent foramen ovale (PFO) (9) in patients with MA compared with controls led to the hypothesis that PFO might play a role in MA pathogenesis and may be a common cardio-embolism source in MA patients with stroke. Indeed, patients with MA most often suffer from cryptogenic stroke, independent of PFO presence (10, 11).

In contrast, the clinical impact of atrial fibrillation has still to be clearly established (12, 13). An impairment in cerebral hemodynamics, either constitutional or as the result of recurrent attacks or triptan intake, has often been claimed as another potential determinant of stroke in patients with migraine (14). Nevertheless, as an apparent paradox, patients with MA tend to have more reactive inter-ictal cerebral hemodynamics, at least in the anterior circulation (15, 16) and, with some reports, also in the posterior circulation (14). Along the same line, the lack of traditional vascular risk factors (17) and large artery atherosclerosis characterize migraine patients with stroke (18, 19).

The carotid wall [i.e., intima–media thickness (IMT)] is influenced by endothelial health. Local environment changes induce the endothelium to secrete several substances, including endothelin-1 (ET-1), von Willebrand Factor (vWF), plasminogen activator inhibitor-1, and platelet activation. These can result in local inflammation and thrombosis, a phenomenon defined as endothelial activation (7). Platelet activation induces augmented aggregation and interaction with leucocytes, which have been observed in migraine (20). Patients with migraine also present a pro-inflammatory and pro-coagulative milieu (21, 22) that justifies the exponential effect that smoking and estrogens exert on stroke risk in MA (23).

Cohort studies addressing carotid IMT consistently reported higher values in patients with migraine with or without aura than controls (24–26).

This study investigates subclinical and clinical large vessel atherosclerosis in stroke patients with MA compared with other young and middle-aged stroke patients without MA.

## Methods

### Participants and study design

This multicenter cohort study was conducted in four hospitals in Northern and Central Italy: ASST Grande Ospedale Metropolitano Niguarda in Milan, ASST Bergamo Est in Seriate (Bergamo), Marche Polytechnic University Hospital in Ancona, and Foundation Campus Bio-Medico University Hospital in Rome.

Our primary endpoints were to investigate whether stroke patients with MA (MA+) present a different burden of subclinical and clinical atherosclerosis than those without MA (MA–).

We retrospectively analyzed the prospectively collected electronic clinical dossiers of consecutive patients discharged from our hospitals with the diagnosis of acute ischemic stroke from January 2016 to June 2022.

Inclusion criteria:

a. clinical diagnosis of acute ischemic stroke confirmed by radiological findings;
b. age 18 years or older and younger than 60 years.

Exclusion criteria: Clinical manifestations lasting longer than 24 h but with negative radiologic findings were excluded to avoid stroke mimics confounders.

### Clinical assessment

All patients received diagnostic and therapeutic care according to the Italian Stroke Association guidelines (https://isa-aii.com/). Stroke was defined as focal neurological symptoms associated with radiological findings (either MR or CT) of acute cerebral ischemia. Migraine with aura and migrainous infarction was diagnosed according to the International Headache Society classification (2).

Our hospitals were provided with an electronic clinical dossier where patient medical history is recorded according to a semi-structured clinical chart model. All patients were interviewed (with the help of family members in case of aphasia or impaired alertness) according to a semi-structured clinical chart model. In MA+ patients, characteristics of aura were also collected: (a) type (pure visual compared to non-visual or multimodal), (b) frequency (i.e., number of episodes) in the last year, (c) the usual duration of the aura phenomenon, and (d) the onset age of MA.

### Vascular risk factors

Hypertension was defined as a history of high blood pressure, systolic blood pressure of ≥140 mmHg, diastolic blood pressure of ≥90 mmHg, or the use of antihypertensive therapies before stroke onset. Dyslipidemia was defined as a history of dyslipidemia, fasting serum total cholesterol level of ≥6.22 mmol/L (2.4 g/L), a triglyceride level of ≥2.26 mmol/L (2 g/L), or the use of statins or fibrates before stroke onset. Diabetes mellitus was defined as a previous diagnosis of type 1 or type 2 diabetes, a history of diabetes mellitus, a fasting serum glucose level of ≥7.0 mmol/L (1.26 g/L), or using an oral antihyperglycemic or insulin before stroke onset. Smoking was defined as active tobacco smoking. The active use of recreational drugs and estrogenic-progestin therapy was also collected. Seventy-two-hour electrocardiographic monitoring or 24 h Holter electrocardiography in case of negative findings was performed to detect atrial fibrillation or other arrhythmias. In selected patients, prolonged non-invasive or invasive EKG monitoring was carried out. We defined the presence of autoimmunity as a positive finding of autoantibodies against extractable nuclear antigens (ENAs) and/or antinuclear antibodies (ANAs) analyzed during the hospital stay.

### Large vessel atherosclerosis and carotid IMT

Carotid and vertebral artery atherosclerosis were investigated with ultrasound duplex as described elsewhere (27). Briefly, the neck vessel arteries were assessed by continuous-wave Doppler and color flow B-mode Doppler ultrasound using high-resolution 7.5 MHz transducers (Philips iU22, Bothell, WA, USA). The best images were digitized and stored for central reading and interpretation. The degree of carotid stenosis was established by means of combined criteria considering blood flow velocities as well as morphological characteristics (28). According to the Mannheim consensus, a carotid plaque was defined as a focal structure protruding into the arterial lumen of at least 0.5 mm or 50% of the surrounding IMT value or showing a thickness of >1.5 mm measured from the media–adventitia interface to the intima–lumen interface (29). For each segment, a plaque scoring system was set as follows: 0, no plaque; 1, small plaque, i.e., stenosis <30%; 2, mild stenosis, i.e., (range 30–49%); 3, moderate stenosis (range 50–69%); and 4, severe stenosis or occlusion (≥70%). “High-risk” plaque was defined as grades 3 and 4 and/or with the morphological characteristics of instability (i.e., echolucency and ulcer) (30). We named “symptomatic large vessel stenosis” the presence of carotid “high-risk” plaques or vertebral or intracranial stenosis in the territory of the ischemic stroke while “clinical atherosclerosis” the presence of carotid stenosis of at least a moderate degree, and/or any other vertebral or intracranial stenosis in any vascular territory.

Neck vessel stenosis >50% or occlusion was confirmed with magnetic resonance imaging (MRI) and/or computed tomography (CT) angiography.

Carotid IMT was measured as described elsewhere according to the Mannheim consensus (27, 29) in Ancona, Bergamo, and Roma hospitals. Interrater reproducibility has been previously assessed in the Ancona and Rome ultrasound labs (31). We considered right and left IMT and the mean value of both sides (mean IMT).

Intracranial stenoses were investigated with MRI and/or CT angiography and transcranial ultrasound duplex as complimentary evaluation.

### Patent foramen ovale

Transthoracic echocardiography (TTE) was performed in all patients. In patients without a definite cause of stroke, upon clinical indication, transcranial Doppler and/or transesophageal echocardiography (TEE) (at rest and during provocative maneuvers using IV injection of agitated saline) were performed to detect PFO and septal interatrial aneurysm (SIA) and other potential cardiac sources of embolism. The presence of PFO with SIA and large PFO (>30 bubbles at rest) was classified as high-risk PFO (32). Further investigations (e.g., screening for acquired or genetic thrombophilia and homocysteine blood levels) were performed upon clinical indication. The stroke severity at admission was assessed by the National Institutes of Health Stroke Scale (NIHSS) (http://nihstrokescale.org). Stroke etiology was defined in all patients according to the TOAST classification (33).

The study received approval from the local Ethical Committee (Prot: 17/20 OSS ComEt CBM), which waived participants' need for written consent because of the retrospective study design and the anonymous data set. Anonymized data will be shared by request from any qualified investigator.

### Statistical analysis

No sample size calculation was performed as this convenience sample was based on the available data. Statistical analyses were performed with SPSS version 28.0 (SPSS Inc., Chicago, IL, USA). The interval variables between groups were compared with independent *t*-test (expressed as means with standard deviations [SD]) or Mann–Whitney U-tests (medians with interquartile range [IQR]) according to data distribution assessed with contingency tables (chi-square and two-tailed Fisher exact tests), and unadjusted odds ratios (ORs) with their 95% confidence intervals (CIs) were run to compare frequencies between groups. All tests were two-tailed. Statistical significance was set with a *p*-value of < 0.05.

Thereafter, we ran forced entry binary logistic regression to adjust risk factors for age and sex and investigated if MA was correlated with IMT and large vessel atherosclerosis. Subjects with missing information on extracranial or intracranial atherosclerosis were excluded. Variables were considered for the analyses if available for at least two-thirds of the patients with the exception of IMT. No imputation was done for missing baseline data. Stepwise linear or binary regression analysis was carried out to adjust correlations for sex, age, and vascular risk factors.

## Results

**Figure 1:**
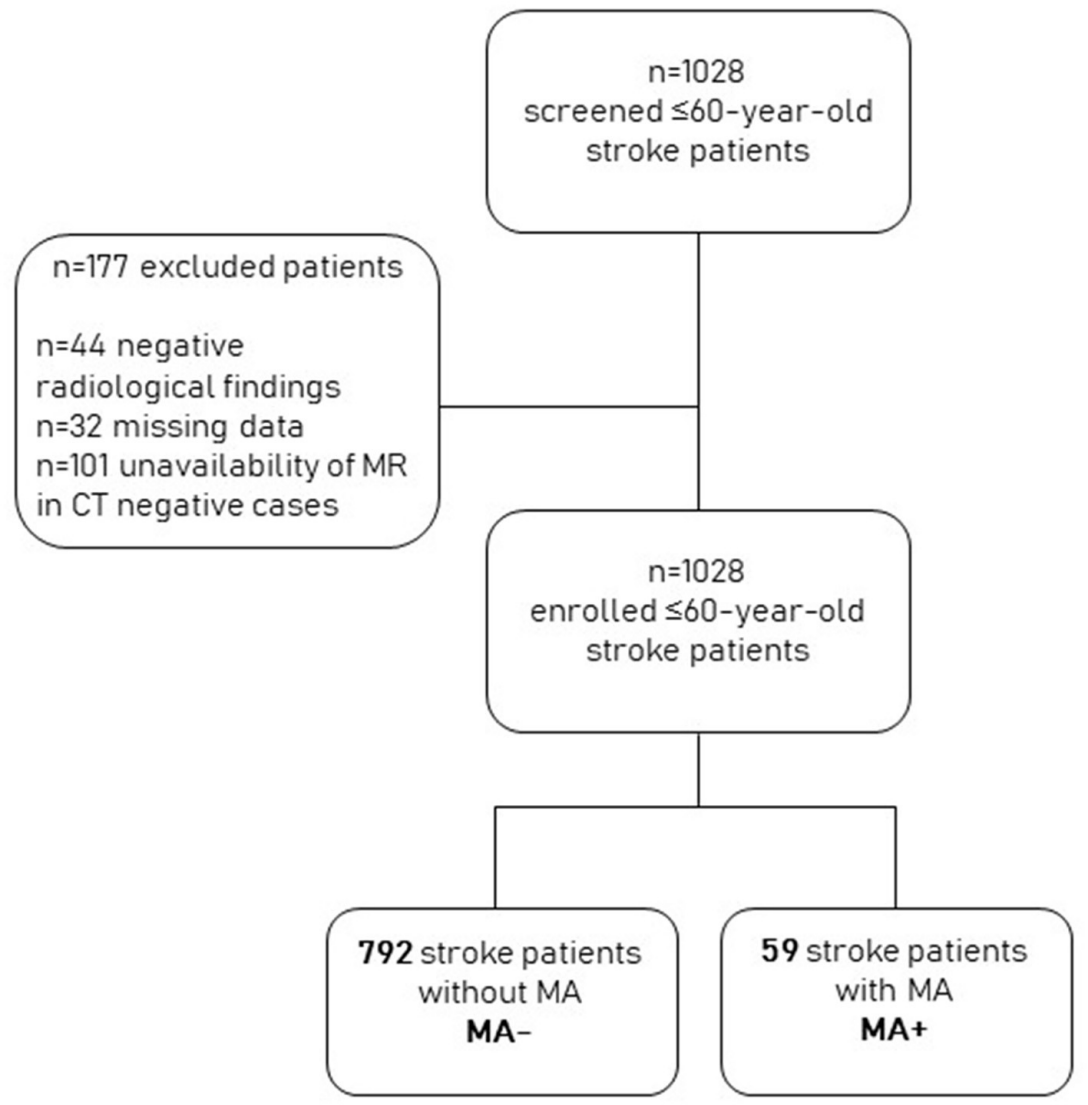
Study population and design.

Of the 1,028 screened patients, 101 were excluded because they had a negative brain CT scan and had not undergone MRI during their hospital stay, 44 because they had negative radiological findings, and 32 because they had missing data. We finally included 851 patients with stroke. Of these, 59 (6.9%) also had an MA diagnosis (MA+). The remaining 792 were classified as MA– (Figure 1).

Table 1 summarizes demographics, clinical variables, and stroke features in MA+ compared to MA– participants. Overall, MA+ participants were younger and characterized by a lower burden of vascular risk factors (diabetes [OR 0.185, 95% C,I 0.045–0.768], hypertension [OR 0.215, 95%CI, 0.107–0.430], and dyslipidemia [OR 0.355, 95%CI, 0.197–0.642]), were mainly female [OR 3.225, 95% CI,1.958–5.310], presented cerebellar stroke more often [OR 3.017, 95% CI, 1.607–5.665], and subcortical infarct less frequently [OR 0.281, 95% CI, 0.163–0.482] compared to MA– participants. After adjusting for age and sex, hypertension (OR 0.349, 95%CI, 0.167–0.470; *p* = 0.005) and dyslipidemia (OR 0.523, 95% CI, 0.280–0.974; *p* = 0.041) but not diabetes (*p* = 0.129) were confirmed as less prevalent in the MA+ group; cerebellar stroke (OR 3.218, 95%CI, 1.657–6.250; *p* ≤ 0.001) showed still distinctive of MA+ patients, while subcortical ischemic lesions were not (*p* = 0.117).

**Table 1:** Comparison of demographics and clinical findings in MA– and MA+ patients.

	**Cohort (*n* = 851)**	**MA– (*n* = 792)**	**MA+ (*n* = 59)**	***p***
Age years, mean (SD)	49.7 (8.5)	50.1 (8.2)	44.0 (10.6)	<0.001
Sex % of female *n* (%)	265 (31.1)	230 (29.0)	35 (59.3)	<0.001
NIHSS median (IQr)	3 (5)	3 (5)	3 (5)	0.284
Stroke territory *n* (%)				0.032
*Anterior*	574 (67.4)	534 (67.4)	40 (67.8)	
ICA	36 (4.2)	35 (4.4)	1 (1.7)	
MCA	528 (62.0)	491 (62.0)	37 (62.7)	
ACA	10 (1.2)	8 (1.0)	2 (3.4)	
*Posterior*	244 (28.7)	225 (28.4)	19 (32.2)	
PCA	68 (8.0)	67 (8.5)	1 (1.7)	
VB	176 (20.7)	158 (19.9)	18 (30.5)	
Multiterritory	33 (3.9)	33 (4.2)	0 (0)	
Stroke site *n* (%)				
Cortico-subcortical	406 (47.9)	376 (47.7)	30 (50.8)	0.686
Subcortical	251 (29.6)	241 (30.5)	10 (16.9)	0.027
Cerebellar	95 (11.2)	80 (10.2)	15 (25.4)	0.002
Brainstem	120 (14.2)	114 (14.5)	6 (10.2)	0.700
Smoking habit *n* (%)	384 (45.2)	362 (45.8)	22 (37.3)	0.224
Illicit drug use *n* (%)	32 (3.8)	32 (4.1)	0	0.164
Estrogenic-progestinal therapy %F	52 (19.7)	42 (18.3)	10 (28.6)	0.172
Hypertension *n* (%)	396 (46.5)	386 (48.7)	10 (16.9)	<0.001
Dyslipidemia *n* (%)	419 (49.5)	403 (51.1)	16 (27.1)	<0.001
Diabetes *n* (%)	128 (15.1)	126 (15.9)	2 (3.4)	0.007
AF and high-risk cardiopathies *n* (%)	63 (7, 4)	61 (7.7)	2 (3.4)	0.356

NIHSS, NIH stroke scale; ICA, internal carotid artery; MCA, middle cerebral artery; ACA, anterior cerebral artery; PCA, posterior cerebral artery; VB, vertebral and basilar arteries; AF, atrial fibrillation.

Stroke patients with MA presented visual aura more commonly (55, 88.1%) with a mean duration of 19.8 min (SD 11.6) and a frequency of 11.4 attacks per year (SD 11.7, min 2, max 120). They reported an MA onset age of 26.8 years (SD 12.8), with a mean time interval of 17.3 years (SD 12.9) from the MA onset to stroke occurrence.

Table 2 shows the results of diagnostic workup and stroke classification in MA+ and MA- participants. Carotid IMT was evaluated in 347 subjects (32 MA+, 54.2%, and 315 MA–, 39.8%; *p* = 0.084), and serum homocysteine was analyzed in 548 patients (50 MA+, 84.7% and 498 MA–, 62.9%; P < 0.001).

**Table 2:** Diagnostic workup and stroke classification in MA– and MA+ patients.

	**Cohort (*n* = 851)**	**MA– (*n* = 792)**	**MA+ (*n* = 59)**	***p***
**Carotid findings**				
Right IMT, mean (SD)	0.72 (0.18)	0.73 (0.18)	0.63 (0.14)	0.003
Left IMT, mean (SD)	0.73 (0.21)	0.73 (0.21)	0.65 (0.15)	0.013
Average IMT, mean (SD)	0.73 (0.18)	0.73 (0.18)	0.64 (0.13)	0.005
Ipsilateral carotid stenosis (*n*, %)				0.016
No plaque	534 (62.7)	487 (61.5)	47 (79.6)	
Small plaques	225 (26.4)	216 (27.2)	9 (15.3)	
Mild stenosis	41 (4.8)	40 (5.1)	1 (1.7)	
Moderate stenosis	14 (1.6)	14 (1.8)	0	
Severe stenosis-occlusion	37 (4.3)	35 (4.4)	2 (3.4)	
High-risk plaque	61 (7.2)	59 (7.4)	2 (3.4)	0.115
Ipsilateral vertebral stenosis (*n*, %)	37 (4.3)	37 (4.7)	0	0.168
Symptomatic intracranial stenosis (*n*, %)	70 (8.2)	70 (8.8)	0	0.011
Symptomatic large vessel stenosis (*n*, %)	140 (16.5)	138 (17.4)	2 (3.4)	<0.001
Contralateral high-risk plaque (*n*, %)	28 (3.2)	28 (3.5)	0	0.250
Contralateral vertebral stenosis (*n*, %)	18 (2.1)	18 (2.2)	0	0.624
Asymptomatic intracranial stenosis (*n*, %)	23 (2.7)	23 (2.9)	0	0.157
Clinical atherosclerosis (*n*, %)	159 (18.7)	157 (19.8)	2 (3.4)	<0.001
Positive finding at PFO investigation *n* (%)	188 (47.4)	152 (43.3)	36 (76.6)	<0.001
SIA % *n* (%)	83 (9.8)	71 (9.0)	12 (20.3)	0.010
High-risk PFO *n* (%) of detected PFO	110 (58.8)	92 (60.9)	18 (50.0)	0.261
Homocysteine blood levels mmol/L, mean (SD)	16.1 (12.1)	13.5 (11.8)	18.0 (15.0)	0.351
Autoimmunity *n* (%) of tested cases	70 (23.3)	59 (22.3)	11 (30.6)	0.294
TOAST criteria *n* (%)				<0.001
Large artery	92 (10.8)	92 (11.6)	0	
Small-vessel disease	219 (25.7)	210 (26.5)	9 (15.3)	
Cardioembolic	95 (11.2)	93 (11.7)	2 (3.4)	
Other causes	148 (17.4)	134 (16.9)	14 (23.7)	
CIS:cryptogenic	297 (34.9)	264 (33.2)	34 (57.6)	

IMT, intima–media thickness; PFO, patent foramen ovale; SIA, septum interatrial; TOAST, trial of ORG 10172 in acute stroke treatment.

The clinical investigations to detect PFO were performed in 47 (79.7%) MA+ patients and in 350 (44.2%) MA– patients (*p* < 0.001). Similarly, autoimmunity was investigated more often in MA+ (36, 61%) than in MA– (264, 33.3%) patients (*p* < 0.001).

After adjusting for age and sex, IMT was not different in MA+ compared to MA– participants (*p* = 0.692), being largely correlated with age (*p* ≤ 0.001). Carotid IMT was also correlated with serum homocysteine after correction for age and sex (*p* = 0.041).

MA+patients were more often affected by cryptogenic stroke (OR 3.144, 95% CI, 1.825–5.416, *p* < 0.001) also when adjusted for age and sex (OR 2.594, 95% CI, 1.483–4.537; *p* < 0.001).

Table 3 displays the association between MA and high-risk PFO, carotid IMT, symptomatic large vessel stenosis, and clinical atherosclerosis after adjusting for age, sex, diabetes, hypertension, dyslipidemia, and smoking habit. Cerebellar strokes did not correlate with the presence of high-risk PFO (19.1 vs. 18.8%; *p* = 1.000).

**Table 3:** Association of MA (independent variable) with high-risk PFO, carotid IMT, symptomatic large vessel stenosis, and clinical atherosclerosis (dependent variables) after adjusting for age and sex, diabetes, hypertension, dyslipidemia, and smoking habit.

	**B**	**OR**	**95% CI**		***p***
			**Lower**	**Upper**	
Migraine with aura	Average IMT				
	−0.27	0.032	−0.090	0.035	0.395
	Symptomatic large vessel stenosis				
	−2.075	0.126	0.017	0.924	0.042
	Clinical atherosclerosis				
	−2.176	0.103	0.014	0.761	0.026
	High-risk PFO				
	−0.247	0.781	0.390	1.564	0.485

## Discussion

Migraine with aura is one of the most common stroke mimics that clinicians deal with in emergency settings (34). Indeed, migraine, especially with aura, increases stroke risk, and given its high prevalence, it should be given more consideration in vascular prevention programs. In this light, it is of pivotal importance to fully understand the pathological mechanisms subtending stroke in patients with migraine. On the one hand, it would allow for the implementation of tailored effective stroke prevention strategies; on the other hand, it would increase the knowledge of migraine physiopathology.

The findings of this study outline a peculiar profile of patients with stroke with MA. In our cohort, patients with MA were younger, more often female, and had fewer vascular risk factors (i.e., diabetes and dyslipidemia) even when adjusted for sex and age. We also observed a negative association between symptomatic large vessel stenosis, any relevant atherosclerosis, and MA diagnosis. This result suggests that the lack of large vessel atherogenesis is a distinctive feature of patients with MA with stroke and confirms earlier reports drawing similar conclusions in cohorts with different age ranges (18, 19, 35, 36). Our study also addressed subclinical atherosclerosis. Carotid IMT is a marker of endothelial activation leading to atherosclerosis and is associated with stroke risk, plaque progression, and neurodegenerative disorders (27, 30, 37). Carotid IMT is also largely dependent on inflammation and oxidative processes (38). Cohort studies have shown that patients with migraine present intima–media thickening (24–26), and here we confirm that, in contrast to large vessel atherosclerosis, patients with MA present age and sex-adjusted IMT similar to other patients with stroke. This observation highlights the potential pathogenic role of endothelial activation in predisposing patients with migraine to stroke.

The endothelium is an active endocrine and paracrine organ playing a central role in multiple functions. Its release of paracrine substances mediates vascular permeability, vascular tone, local inflammation, and thrombosis. The endothelium homeostasis is kept by balanced actions of substances with opposing functions. Some of these (e.g., nitric oxide, ET-1, vWF, platelet-activating factor, and homocysteine) have been consistently implied in migraine pathogenesis, especially in MA (7, 22). If the endothelium is damaged by mechanisms related to vascular risk factors or genetic or exogenous factors, this balance is lost, resulting in the so-called endothelial activation. To note, in our cohort serum, homocysteine was correlated with IMT after age and sex adjustment. The detrimental relationship between endothelial activation and migraine is probably bidirectional. On the one side, the unbalanced release of endothelial substances and microcirculation thrombosis can trigger cortical spreading depression (39); on the other side, frequent migraine attacks seem to interfere with endothelial functioning (26). In any case, the cerebral cortex of patients with MA seems to be particularly vulnerable to ischemic insults, displaying larger infarct volume (40) and a reduced ratio between hypoperfused and infarcted ischemic areas (41). This sensitivity to ischemic insults can result from different factors, among which the most relevant seems to be an impairment of vascular tone regulation secondary to endothelial dysfunction as an inefficient use of energy supplies (42). The younger age and the lower impact of traditional risk factors in patients with MA in our and other cohorts (19, 34) support these hypotheses.

Our study also observed that MA patients more frequently presented cerebellar stroke than MA– participants. The camera study (43) reported for the first time a higher prevalence of silent ischemic infarct in the posterior circulation (i.e., cerebellum) of patients with migraine, suggesting a combination of (possibly migraine attack-related) hypoperfusion and embolism but not atherosclerosis or small-vessel disease as the possible determinants. More recently, the AGES-Reykjavik study confirmed the higher prevalence of cerebellar infarcts in MA and higher incidence in patients with any migraine (44), as also observed in the acute settings (45).

We report a higher frequency of clinically relevant cerebellar strokes in patients with MA that were not correlated with the presence of high-risk PFO. It should be noted that, while PFOs of any degree were more frequently detected in patients with MA [~50–55% (46)], they were only high-risk PFOs in half of the cases. The prevalence of PFO is also very common in the general population, but it is responsible for ischemic stroke only in a small percentage of cases (32). Moreover, no patients with MA presented the steno-occlusive disease of vertebral and basilar arteries. The cerebellar involvement in migraine is supported by neurophysiological, neuroimaging, and genetic studies (47). Patients with MA can also display impairment in cerebellar circulation autoregulation (48). In this line, cerebellar hypoperfusion and crossed cerebellar diaschisis are common in patients with migraine with aura. However, this phenomenon is usually of benign origin as it is not associated with cerebellar infarctions, even in patients with prolonged symptom-related perfusion abnormalities persisting for up to 24 h (49). Nevertheless, it is possible that under specific conditions, these perfusion alterations produce permanent damage. It should be noted that Purkinje cells are selectively prone to hypoxic insults (50). The brain of patients with MA, which is highly energy-demanding but unable to use energetic supply effectively, can easily subdue the ischemic damage in vulnerable neurons, especially in the presence of altered autoregulation and/or endothelial activation.

The main limitation of our study is the retrospective design. Our structured electronic dossiers, however, enabled us to collect prospectively in detail the studied variables. Another limitation is the lack of identification and characterization of migraine without aura in all stroke subjects. However, when not accompanied by transient focal symptoms, headache is rarely investigated or adequately reported in real-life clinical settings, producing a detection bias. Therefore, we preferred not to consider this aspect as it may have been largely underestimated in the whole cohort.

**Figure 2:**
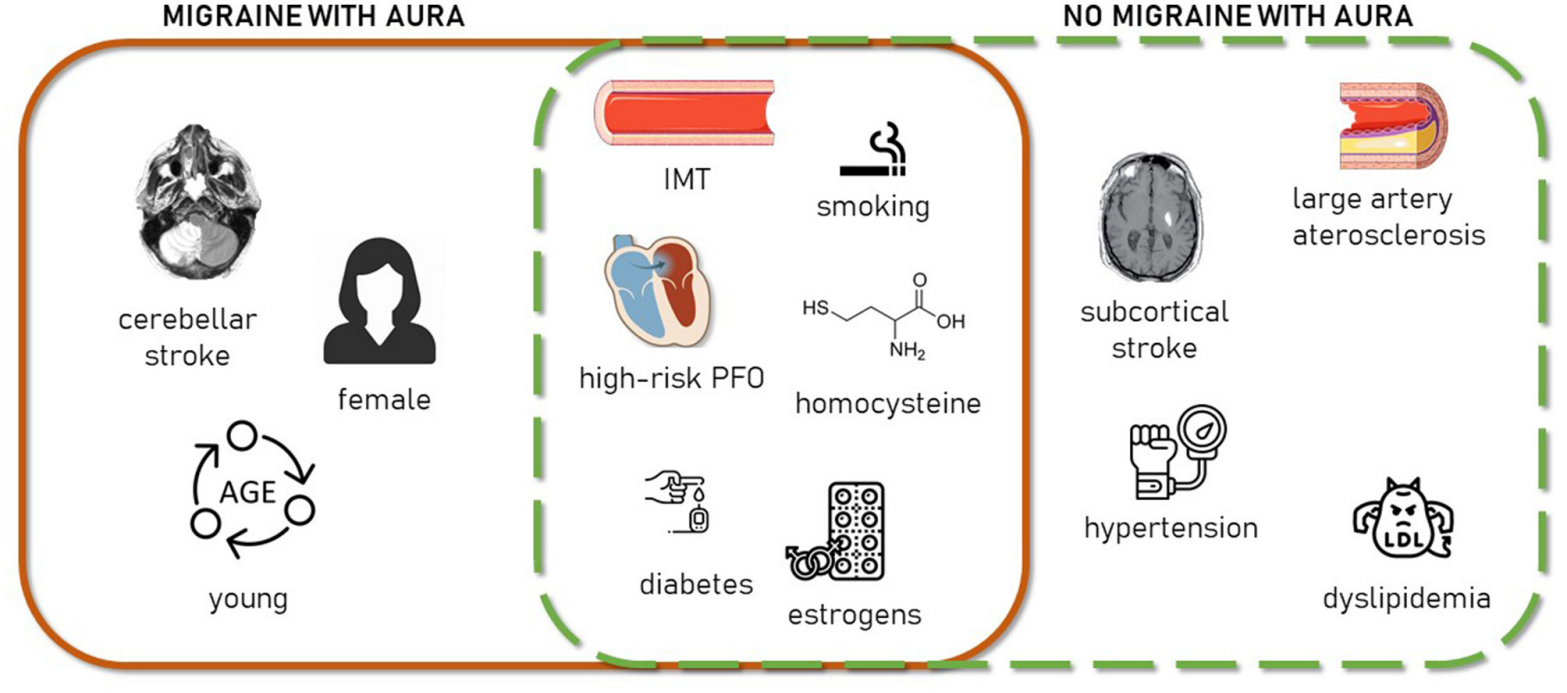
Schematic representation of clinical stroke-related aspects characterizing MA+ patients (continuous line square, left), MA– patients (dashed line square, right), or both (square intersection).

In summary, our findings suggest that young and middle-aged patients with stroke with MA present a peculiar clinical profile characterized by a less prominent role of traditional vascular risk factors and clinical atherosclerosis and by ischemic damage in vulnerable brain areas, probably mediated by endothelial and local hemodynamic impairment and inefficient energetic use (Figure 2).

Our study warrants more research in the field of vascular aspects of migraine as the prevention of common vascular risks is insufficient to address migraine-related stroke risk. This perspective appears even more relevant since the introduction of the new migraine preventive therapies targeting the calcitonin gene-related peptide, one of the most potent vasodilatory peptides in cerebral circulation. Although clinical studies do not support any detrimental impact on cerebral and systemic hemodynamics (51), cautious vigilance is necessary.

## Data availability statement

The raw data supporting the conclusions of this article will be made available by the authors, without undue reservation.

## Ethics statement

The studies involving human participants were reviewed and approved by Campus Bio-Medico University. Written informed consent for participation was not required for this study in accordance with the national legislation and the institutional requirements.

## Author contributions

CA conceptualized the study and analyzed and interpreted the findings. GV, AR, and PM collected the data and interpreted the findings. NB and MM collected the data. VL, FF, EA, MS, and FV interpreted the findings and revised the manuscript for intellectual content. All authors contributed to the article and approved the submitted version.
